# Neuronal CD47 induces behavioral alterations and ameliorates microglial synaptic pruning in wild-type and Alzheimer’s mouse models

**DOI:** 10.1186/s13578-025-01378-x

**Published:** 2025-03-26

**Authors:** Wenjie Hu, Mengting Chen, Yuxue Lin, Hui Zhang, Li Sun, Wei Shao, Yuping Ye, Yujie Cheng, Shanshan Zhou, Panpan Hu, Xingqi Wu, Yin Xu, Kai Wang

**Affiliations:** 1 Department of Neurology, The First Affiliated Hospital of Anhui Medical University, Anhui Medical University, Hefei, 230022 China; 2 School of Mental Health and Psychological Sciences, Anhui Medical University, Hefei, 230032 China; 3 Collaborative Innovation Center of Neuropsychiatric Disorders and Mental Health, Hefei, 230032 China; 4 Anhui Province Key Laboratory of Cognition and Neuropsychiatric Disorders, Hefei, 230032 China; 5 Laboratory of Molecular Neuropsychiatry, Anhui Medical University, Hefei, 230032 China; 6 Anhui Institute of Translational Medicine, Hefei, 230032 China

**Keywords:** CD47, Microglia, Alzheimer’s disease, Autism spectrum disorder, Neuron-microglia interactions

## Abstract

**Background:**

Microglia are brain-resident macrophages that play a crucial role in synapse pruning during the development and progression of various neuropsychiatric disorders, including autism spectrum disorder (ASD) and Alzheimer’s disease (AD). Mechanistically, CD47 protein acts as a potent ‘do not eat me’ signal, protecting synapses from phagocytosis by microglia. However, the functional role of the upregulated neuronal CD47 signal under both physiological and pathological conditions remains unclear.

**Results:**

We utilized an adeno-associated virus gene expression system to induce neuron-specific overexpression of CD47 in wild-type and 5xFAD mice, assessing its effects on microglial synaptic phagocytosis and mouse behaviors. Our results indicate that neuronal CD47 induces ASD-like behaviors and synaptic pruning defects, while promoting behavioral disinhibition and improving memory in wild-type mice. Single-nucleus RNA sequencing was employed to profile gene expression patterns in subpopulations of neurons and microglia. Notably, neuronal CD47 enhances synaptic pathways in neurons and particularly shifts microglial subpopulations from a disease-associated state to a homeostatic state. Additionally, neuronal CD47 reduces excessive microglial synaptic phagocytosis induced by Aβ pathology in 5xFAD mice.

**Conclusion:**

Our study provides evidence that neuronal CD47 overexpression results in synaptic pruning defects and is involved in the pathogenesis of ASD, while also playing a beneficial role in mitigating excessive synaptic loss in Alzheimer’s disease.

**Supplementary Information:**

The online version contains supplementary material available at 10.1186/s13578-025-01378-x.

## Introduction

Microglia, the resident immune cells of the brain play a critical role in maintaining the homeostasis of the central nervous system. Through constant surveillance of inter- and extra-neuronal elements, microglia establish continuous crosstalk with neurons, which is essential for their functions of microglia under physiological and pathological conditions [1, 2]. Recent studies have shown that microglia are required for synaptic remodeling during early development through a process known as synaptic pruning via phagocytosis [3]. Mechanistically, the complement cascade components C1q and C3 localize to pruning sites as “eat me” signals to promote microglial engulfment. Animal studies have demonstrated that mice lacking C1q or C3 fail to eliminate synapses at the early stages of development [4–6]. In contrast, other classes of molecules, such as CD47, called “do not eat me” signals, prevent certain synaptic elements from being targeted by microglia. *Cd47* knockout mice exhibit redundant microglial engulfment and increased functional pruning [7].

Both human and animal studies have also demonstrated possible links between synaptic pruning dysfunctions by microglia and the mechanisms of neuropsychiatric disorders [8–14], particularly autism spectrum disorder (ASD) [15] and Alzheimer’s disease (AD) [16, 17]. In ASD, an inefficient pruning process leads to redundant neuronal connections, causing abnormal behaviors such as social deficits and stereotypical behavior [18]. In contrast, excessive pruning along with microgliosis caused by amyloid β (Aβ) deposition contributes to synaptic loss, and memory and cognitive impairments in AD [19, 20]. Mechanistically, the expansion of specific subpopulations of microglia has been identified in mouse models with Aβ pathology, which are likely phagocytic, such as disease-associated microglia (DAM) [20] or microglial neurodegenerative phenotype (MGnD) [21]. However, the precise regulatory signals for DAM from neurons and involvement of DAM signal in ASD are not well understood.

Here we examined the function of neuronal CD47 in wild-type mice and the 5xFAD mouse model that develops severe Aβ pathology. We report that neuronal CD47 overexpression leads to profound behavioral alterations, including social deficits, stereotypical behavior, and improved memory in wild-type mice. Notably, neuronal CD47 causes behavioral disinhibition, which is characterized by reduced anxiety-like behaviors and increased exploratory activity compared to wild-type mice. At the molecular level, neuronal CD47 overexpression switches microglial subpopulations from a DAM-like state to a homeostatic state and reduces microglial synaptic pruning. Furthermore, neuronal CD47 overexpression significantly reverses excessive microglial phagocytosis and synaptic loss in 5xFAD mice.

## Materials and methods

### Animals

All animals used in this study were bred and housed in exhaust ventilation central cages with five mice per cage at Anhui Medical University. Mice were housed in a regulated environment (the temperature was controlled at 20–23 °C, the humidity was controlled at 40–70%, and the light and dark alternated every 12 h) and had free access to food and water. All procedures involving mice were approved by the Institutional Animal Care and Use Committee of Anhui Medical University. C57BL/6 mice were purchased from the Animal Experimental Center of Anhui Medical University (China). The 5xFAD transgenic mice on the C57BL/6 background were obtained from the Shanghai Model Organisms Center, Inc (China), and were crossed with the C57BL/6 mice for propagation. Both male and female mice were randomly selected for the study.

### In vivo gene delivery

The AAV2/9-hSyn-CD47-Flag-WPRE-hGH poly or AAV2/9-hSyn-mChery-WPRE-hGH PA were produced by BrainVTA (Wuhan, China). The mice were anesthetized with isoflurane and placed on a heating pad to maintain body temperature at 37 °C. Under sterile surgical conditions, the mice were fixed in a stereotaxic instrument (RWD Life Science Co., Ltd.) with eye protection by ointment. For P0 injection as previously described [22], each mouse was injected into the lateral ventricles of both cerebral hemispheres with 5 × 10^10^ total viral particles per side. All mice were euthanized at 4 months of age.

### Three-chamber test

The apparatus is a rectangular device (60 cm × 40 cm × 20 cm) and separated into three equally sized areas by two transparent walls(with transparent door), the mice could walk among the three chambers. For the initial phase, mice were placed in the central chamber and acclimatized for 5 min, then the doors were removed and mice were allowed to freely explore the three chambers for 10 min. In the second phase, assessing sociability. A stranger mouse(Stranger 1) was placed in a metal cage and an empty cage was placed in the opposite chamber. The mice explore freely for 10 min, during which videotaping records interaction time. In the third phase, assessing social novelty, a second stranger mouse(Stranger 2) was placed in another empty metal cage and was allowed 10 min to interact with both Stranger 1 and Stranger 2.

### Grooming test

The experimental mice were placed in a rectangular box (40 cm × 34 cm × 40 cm) and allowed to acclimate for 10 min. Subsequently, an infrared camera was used to record the mice’s self-grooming behavior in the dark for an additional 10 min. The self-grooming behaviors observed included licking of the body and hair, rubbing the face with the front paws, and scratching the trunk.

### Marble burying test

To initiate the experiment, a rearing box measuring 40 cm x 30 cm x 22 cm is prepared, ensuring it is both dry and clean. A bedding layer 5 cm thick is placed at the bottom of the box. Subsequently, 20 standard glass beads, each measuring 14 mm to 15 mm in diameter, are arranged uniformly in 4 columns and 5 rows atop the bedding. Mice are gently introduced into the same corner of the box, and their behavior is observed over a 30-minute period. A glass bead is deemed buried if more than two-thirds of it is covered by the bedding. The number of buried glass beads is recorded for each mouse, with a higher number indicative of increased stereotypical behavior.

### Open field test

The open field test (OFT) is based on the animals’ conflicting innate tendencies to avoid open spaces and explore novel environments. Mice were placed in the center of a brightly lit arena with white walls (50 cm ×50 cm × 40 cm; RWD Life Science Co., Ltd) and allowed to explore freely for 10 min. The animal’s position and movement were detected by a grid of photocells covering the arena, and the behavior was scored automatically using Smart 3.0 software (Panlab S.L.U).

### Elevated plus maze

Elevated plus maze (EPM) consisted of two opposite closed arms measuring 50 cm by 10 cm with enclosed side and end walls of 40 cm high, two open arms and an opened central square measuring 10 cm by 10 cm, were used for this study. Each mouse was placed in the central area facing a closed arm and roamed in the maze for 5 min. Smart 3.0 software (Panlab S.L.U) was used for video-tracking.

### Morris water maze

Morris water maze (MWM) was performed in a circular pool with a diameter of 120 cm and an image capture camera as described previously [23]. The water temperature was kept at 22 ± 1 °C and non-toxic titanium dioxide was added to dye the water a white background. Mice were trained for 4 days with 4 trials per day and 60 s per trial. On the fifth day of the experiment, the platform was removed and all mice were allowed to explore freely for 60 s. The whole process is captured in real-time using Smart 3.0 software (Panlab S.L.U).

### Western blot

Mouse brain tissue was lysed with RIPA lysis buffer containing protease inhibitors and phosphatase inhibitors and centrifuged for 10 min at 4 °C at 12,000 rpm, after which the protein concentration was determined using a BCA kit. The protein samples were separated by sodium dodecyl sulfate-polyacrylamide gel electrophoresis (SDS-PAGE) and then transferred to a polyvinylidene fluoride (PVDF) membrane. Subsequently, the membrane was blocked in 5% buttermilk and incubated at 4 °C with primary antibody overnight. The next day, the membrane was incubated with the secondary antibody at room temperature for 2 h. Finally, the protein bands were visualized using an enhanced chemiluminescence kit. ImageJ software version 2.0 was used to analyze the images on the film.

### Immunofluorescence

For mouse brain analysis, the mice were perfused transcardially with 4% PFA under sodium pentobarbital anesthesia. The brain tissue was immobilized in 4% PFA and transferred to 30% sucrose solution until it was sliced. Coronal brain Sect. (30 μm) were cut on a cryotome and stored in a cryoprotectant at -20 °C. After washing with PBS three times, the membrane was permeated with PBS containing 0.4% Triton X-100 for 30 min, and then blocked with PBS containing 5% BSA for 2 h. Then, the brain sections were incubated at 4 °C with primary antibody overnight. The next day, the slices were washed 3 times in PBST, and incubated with the appropriate secondary antibody at room temperature for 2 h. After washing with PBS, the sections were incubated with DAPI to stain the nucleus. Images were captured using an EVOS microscope (M7000) or confocal microscope (LSM800) and quantified using ImageJ and Imaris software.

### Antibodies

All antibodies used for western blotting and immunofluorescence staining were purchased from commercial sources, as described in Supplementary Table S1.

### Single nucleus RNA sequencing analysis

At 4 months of age, all groups of mice were perfused transcardially with cold PBS. Mouse hippocampal tissue was isolated in an RNase free tissue cryopreservation tube, and a single nuclear suspension was prepared after rapid freezing with liquid nitrogen. The single nucleus RNA sequencing analysis was described in Supplementary Methods. Briefly, nuclear suspensions were loaded on a 10× Genomics GemCode single-cell instrument that generates single-cell Gel Bead-In-EMlusion (GEMs) plates. Libraries were generated and sequenced from the cDNAs with Chromium Next GEM Single Cell 3’ Reagent Kits v3. Bead-In-EMlusion (GEMs). Libraries were generated and sequenced from the cDNAs with Chromium Next GEM Single Cell 3’ Reagent Kits v3. The double-ended sequencing mode of the Illumina sequencing platform was used for high-throughput sequencing of the constructed library. Seurat implements a graph-based clustering approach, and the log-normalized matrices were then loaded on SingleR R packages for cell type annotation, which is based on correlating gene expression of reference cell types with single-cell expression. The expression value of each gene in the given cluster was compared against that of the remaining cells using the Wilcoxon rank sum test. Significantly upregulated genes were identified using a number of criteria. First, genes had to be at least 1.28-fold overexpressed in the target cluster. Second, genes had to be expressed in more than 25% of the cells belonging to the target cluster. Third, the p value was less than 0.05.

### Statistics

The statistical methods used for single nuclear RNA-seq are described in the previous section. The data are presented as the average ± standard error of the mean (SEM). Violin plots are presented as medians and quartiles. Pairwise comparisons were analyzed using a two-tailed Student’s t-test. The statistical analyses were performed using GraphPad Prism software. Values of *P* ≤ 0.05 were considered to indicate a statistically significant difference.

## Results

### Neuronal CD47 overexpression promotes ASD-like behaviors, behavioral inhibition, and memory performance

CD47 expression has been linked to synaptic refinement and various behavioral and cognitive disorders. Notably, high levels of CD47 expression have been associated with abnormal brain overgrowth and the 16p11.2 deletion, which is linked to ASD [24]. Therefore, we hypothesized that CD47 overexpression leads to ASD-like behaviors. To test this, we administered intracerebroventricular injections of AAV-mCherry or AAV-CD47, driven by the synapsin 1 gene promoter, in two groups of wild-type C57/BL6 mice at postnatal day 0 (P0) to achieve neuron-specific gene overexpression [25]. Immunofluorescence staining confirmed significantly greater CD47 expression in the neurons of AAV-CD47 injected mice than AAV-mCherry controls (Fig. S1, A-D).

**Fig. 1:**
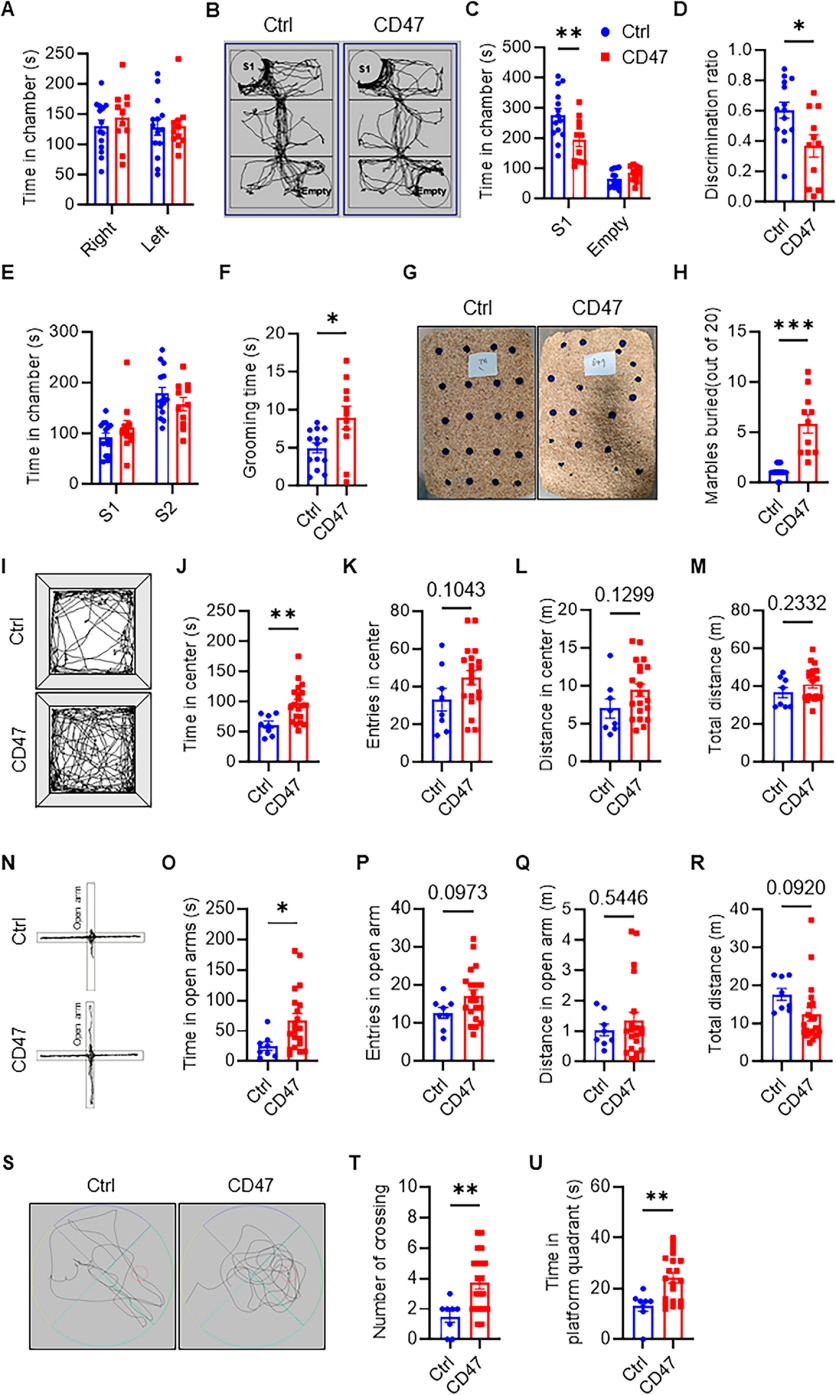
Behavior profiling in wild-type mice with neuronal CD47 overexpression. **(A)** Time spent in the right and left chambers during the habituation phase. **(B)** A representative trajectory plot of control and neuronal CD47 overexpression mice in the three-chamber test. **(C)** Time spent in the right and left chambers during the social phase. **(D)** The discrimination ratio was calculated using the formula: (time in social chamber - time in nonsocial chamber) / total time. (*n* = 14 for control, *n* = 11 for CD47 overexpression mice). **(E)** Time spent in the left and right chambers during the social novelty phase. **(F)** The duration of self-grooming behavior displayed by control and CD47 overexpression mice over a 10-minute period. (*n* = 14 for control, *n* = 11 for CD47 overexpression mice). **(G)** A representative image of buried marbles from the marble burying test. **(H)** The number of marbles buried by control and CD47 overexpression mice over a 30-minute period. (*n* = 14 for control, *n* = 11 for CD47 overexpression mice). **(I)** A representative trajectory plot of control and neuronal CD47 overexpression mice in the open field test. **(J-M)** Time **(J)**, number of entries **(K)**, and distance traveled **(L)** in the inner zones of the open field arena. **(M)** Total distance traveled in the inner and outer zones of the open field arena (*n* = 8 for control, *n* = 20 for CD47 overexpression mice). **(N)** A representative trajectory plot of control and neuronal CD47 overexpression mice in the elevated plus maze. **(O-R)** Time **(O)**, number of entries **(P)**, and distance traveled **(Q)** in the open arm of the elevated plus maze. **(R)** Total distance traveled in the open and closed arms of the elevated plus maze (*n* = 8 for control, *n* = 20 for CD47 overexpression mice). **(S)** A representative trajectory plot of control and neuronal CD47 overexpression mice in the Morris water maze. **(T-U)** Number of platform crossings **(T)** and time traveled **(U)** in the target quadrants of the Morris water maze (*n* = 8 for control, *n* = 20 for CD47 overexpression mice). Data are presented as the mean ± SEM. ^*^P ≤ 0.05, ^**^P ≤ 0.01

We then assessed the social behavior of CD47-overexpressing mice at 2 months of age using the three-chamber test. During the habituation phase, neither group showed a preference for either chamber (Fig. 1A). When an unfamiliar mouse (Stranger 1) was introduced in one chamber with the other chamber remaining empty, CD47-overexpressing mice spent significantly less time interacting with the unfamiliar mouse than controls (Fig. 1B-D), suggesting impaired social abilities. In addition, we evaluated social novelty by introducing a second unfamiliar mouse (Stranger 2) into the empty chamber. CD47-overexpressing mice did not show significant differences in time spent interacting with Stranger 2 compared to Stranger 1 (Fig. 1E), indicating that the social deficits observed are primarily related to social interaction rather than novelty recognition. Repetitive stereotypical behavior, another ASD core symptom, was assessed through grooming and marble burying tests. CD47 mice groomed more frequently (Fig. 1F), and buried more marbles than control mice (Fig. 1G-H). Combined, these findings indicate that neuronal CD47 overexpression induces ASD-like behaviors in wild-type mice.

To further investigate alterations, we utilized the open field test to assess anxiety and locomotor behaviors [25]. There were no significant differences observed in the number of entries (Fig. 1K) or the distance traveled (Fig. 1L) in the center of the open field arena between CD47-overexpressing and control mice, indicating no changes in general mobility or motor function. Notably, CD47-overexpressing mice spent more time in the center of the open field arena (Fig. 1I, J), suggesting behavioral disinhibition and reduced anxiety. Consistently, these mice also spent more time in the open arms of the elevated plus maze (Fig. N-O), which is another assay for anxiety-related behavior [26]. Thus, CD47 overexpression results in specific behavioral disinhibition and decreased anxiety.

To explore the role of CD47 in spatial learning and memory, we conducted the Morris water maze test [23]. CD47-overexpressing mice performed better in the spatial probe test, as indicated by a significantly increased number of platform crossings (Fig. 1S-T) and time in the platform quadrant (Fig. 1U). Collectively, these findings demonstrate that neuronal CD47 overexpression enhances behavioral disinhibition and memory performance in wild-type mice.

### Neuronal CD47 overexpression inhibits microglial phagocytosis and synaptic pruning

**Fig. 2:**
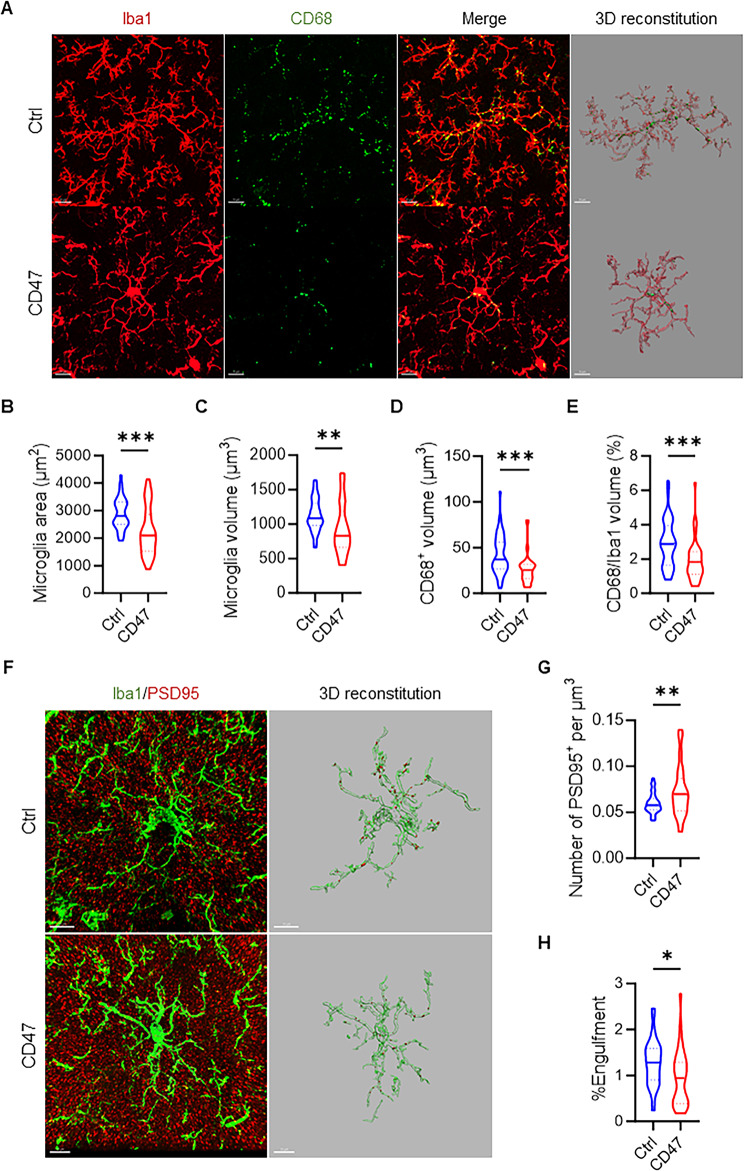
Neuronal CD47 overexpression leads to excessive synapses. **(A)** Representative images of Iba1 and CD68 co-immunostaining, along with 3D renderings, in the hippocampal CA3 region of control and neuronal CD47-overexpression mice at 4 months of age. Scale bar: 10µM. **(B-C)** Quantification of the surface area **(B)** and cellular volume **(C)** of microglia in the hippocampus of control and neuronal CD47 overexpression mice. **(D-E)** Quantification of CD68-positive volume **(D)** and the percentage of CD68-positive volume within microglia **(E)**. (*n* = 4 for control, *n* = 5 for CD47 overexpression mice). **(F)** Representative images of Iba1 and PSD95 co-immunostaining, along with 3D renderings, in the hippocampal CA3 region of control and neuronal CD47-overexpression mice at 4 months of age. Scale bar: 10µM. **(G-H)** Quantification of the number of PSD95 puncta **(G)** and the percentage of PSD95-positive volume within microglia **(H)**. (*n* = 4 for control, *n* = 5 for CD47 overexpression mice). Data are presented as the mean ± SEM. ^*^P ≤ 0.05, ^**^P ≤ 0.01, ^***^P ≤ 0.001

Given that microglia-mediated synaptic dysregulation is implicated in ASD pathogenesis, we examined microglia morphology and phagocytic capacity in the hippocampal CA3 region of 4-month-old CD47-overexpressing and control mice via immunofluorescence assays. Results showed reduced microglial volume (Fig. 2A-B) and surface area (Fig. 2A-C), along with lower microglial phagocytic marker CD68 levels in CD47-overexpressing mice (Fig. 2A, D-E). These mice also had fewer PSD95-positive puncta within microglia (Fig. 2F, H), yet showed increased total PSD95-positive signals in the hippocampus (Fig. 2F-G). These findings highlight that CD47 overexpression diminishes microglia-mediated synaptic pruning.

### snRNA-seq revealed synaptic profiles in mice with neuronal CD47 overexpression

**Fig. 3:**
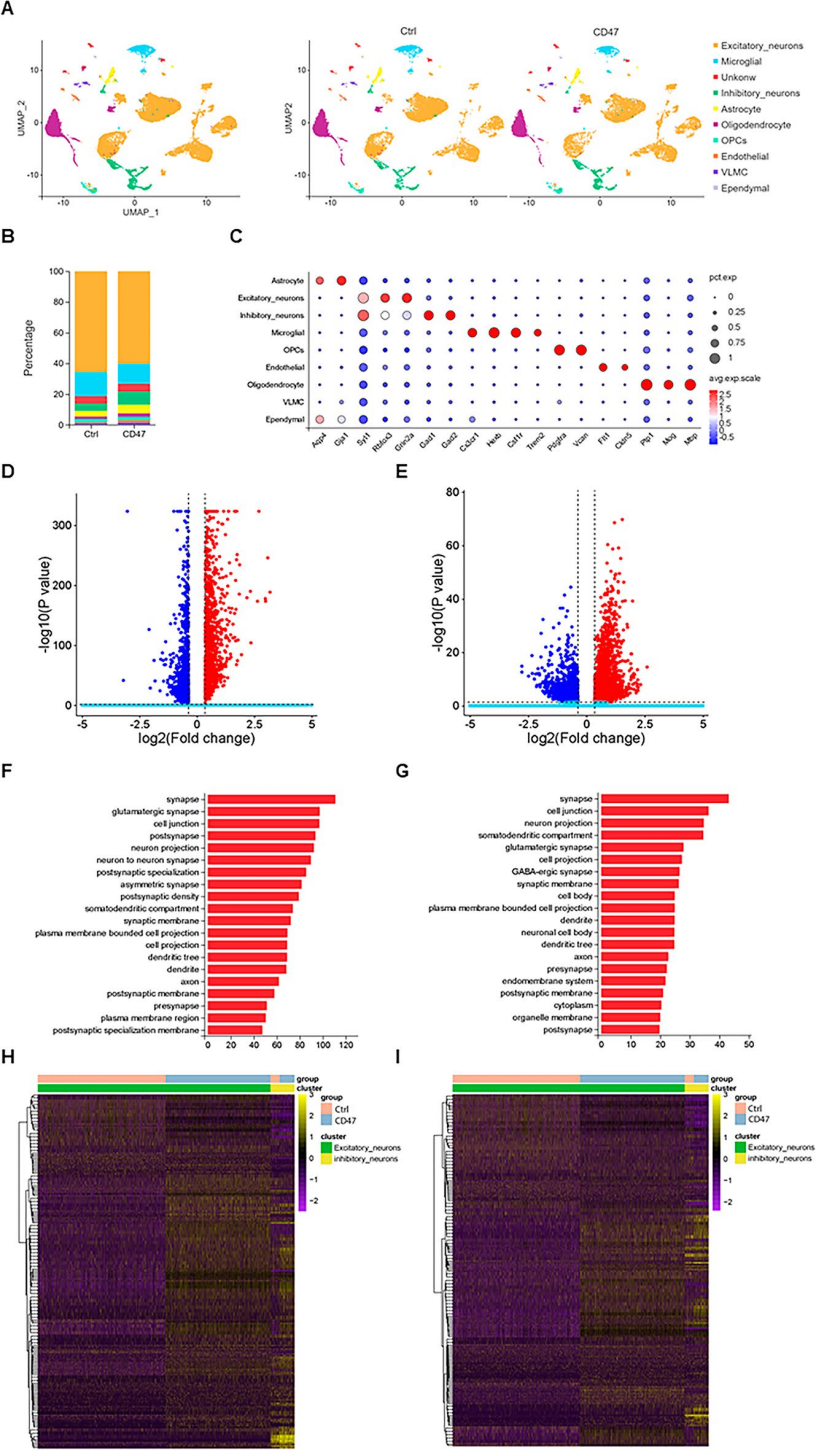
snRNA-seq revealed synaptic pathways in neurons from CD47-overexpression mice. **(A)** Uniform Manifold Approximation and Projection (UMAP) representation of the snRNA-seq data of 21,721 cells from the hippocampus of control and CD47 overexpression mice (left panel) and across groups (right panel). **(B)** Stacked bar graph showing cell-type compositions comparing control and CD47-overexpression mice. **(C)** Average expression levels of selected signature genes for different cell types. **(D-E)**. Volcano plot showing 2,175 and 3,645 DEGs for excitatory neurons **(D)** and inhibitory neurons **(E)**, respectively, in CD47 overexpression versus control mice. Upregulated genes are shown in red, while downregulated genes are shown in blue. **(F-G)** Gene Ontology (GO) enrichment analysis of biological pathways associated with upregulated genes in excitatory neurons **(F)** and inhibitory neurons **(G)** in CD47 overexpression versus control mice. **(H-I)** Heatmap showing 208 and 210 DEGs for pre-synaptic **(H)** and post-synaptic **(I)** pathways in CD47 overexpression versus control mice, respectively

Having established the behavioral profile of CD47-overexpression mice, we next sought to understand cell-type specific alterations by conducting single-nucleus RNA sequencing (snRNA-seq) of the hippocampus collected from 4-month-old wild-type mice after stereotaxic injection of AAV-CD47 or AAV-mCherry into both hippocampal hemispheres. Cell nuclei were isolated by fluorescence-activated cell sorting and profiled using the droplet-based 10× Genomics platform. After rigorous quality control including doublet removal and normalization (Fig. S2A), we obtained a total of 21,721 high-quality single-nucleus transcriptomes (Table S2), which were annotated into nine major cell types based on the expression of well-established cell-type-specific markers (Fig. 3A-C). CD47 was particularly overexpressed in excitatory and inhibitory neurons indicated by snRNA-seq, while the expression of SIRPα, a CD47 receptor, was not affected (Fig. S2B and Table S3).

Cell-type composition analysis between CD47 overexpression and control mice revealed that certain cell-type populations, such as excitatory neurons (65.63% in control mice and 60.11% in CD47-overexpression mice, Table S4), and inhibitory neurons (4.73% in control mice and 8.59% in CD47-overexpression mice, Table S4), were altered after CD47 neuronal overexpression, which correlated with behavioral alterations. Further analysis revealed 2,175 DEGs (differentially expressed genes) in the excitatory neurons of CD47-overexpression mice compared with control mice, of which 1,114 and 1,016 were significantly upregulated and downregulated respectively with a cutoff of FDR < 0.05 and log2(fold change) ≥ 0.36 (Fig. 3D). There were 3,645 DEGs in the inhibitory neurons of CD47-overexpression mice compared with those in the inhibitory neurons of control mice, of which 2,347 and 1,298 were significantly upregulated and downregulated respectively, with a cutoff of FDR < 0.05 and log2 (fold change) ≥ 0.36 (Fig. 3E). Gene Ontology (GO) pathway analysis of the upregulated genes showed synaptic pathways as top enriched pathways in both excitatory neurons (Fig. 3F) and inhibitory neurons (Fig. 3G) in CD47-overexpression mice. Next, we identified 208 and 210 DEGs that correlated with pre-synapse (Fig. 3H) and post-synapse (Fig. 3I) respectively. Together, these results suggest robust synaptic alterations after CD47 neuronal overexpression.

### Neuronal CD47 overexpression suppresses the DAM-like microglial population

**Fig. 4:**
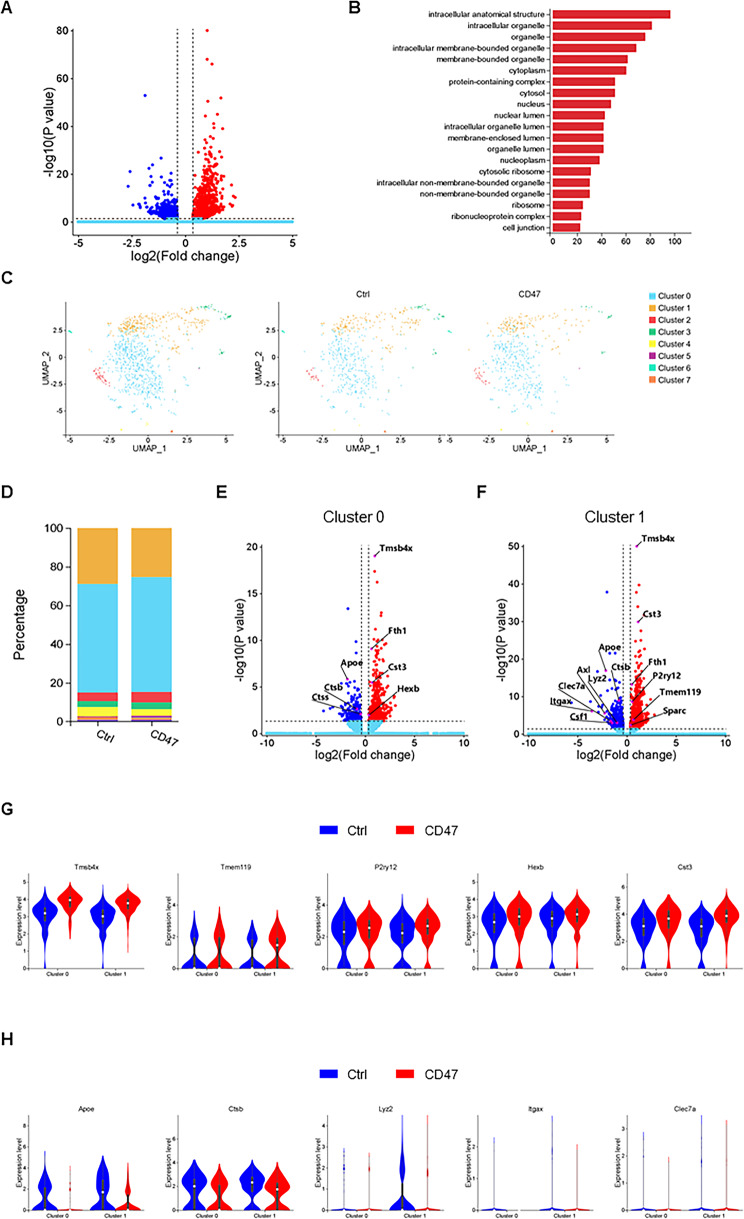
Shift of microglial subpopulations in CD47 overexpression mice. **(A)** Volcano plot showing 1,784 DEGs for microglia in CD47-overexpression versus control mice. **(B)** GO enrichment analysis of biological pathways for upregulated genes in microglia of CD47-overexpression versus control mice. **(C)** UMAP representation of microglia from control and CD47-overexpression mice (left panel) and across groups (right panel). **(D)** Stacked bar graph depicting microglial subpopulation compositions comparing control and CD47-overexpression mice. **(E-F)** Volcano plots showing 775 and 1,475 DEGs for cluster 0 **(E)** and cluster 1 **(F)** microglia in CD47-overexpression versus control mice, respectively. Upregulated genes are shown in red, while downregulated genes are shown in blue. **(G-H)** Upregulated genes are shown in red, while downregulated genes are shown in blue. Violin plots depicting the expression levels of homeostatic and disease-associated microglia (DAM) genes in subcluster 0 and subcluster 1 microglia, respectively

Since CD47 is a key molecule in microglia mediating synaptic remodeling, we wondered whether neuronal CD47 expression level could change microglial status. snRNA-seq identified 1,784 DEGs in the microglial population of CD47-overexpression mice compared with those in the microglial population of control mice, of which 1,000 and 784 were significantly upregulated and downregulated respectively, with a cutoff of FDR < 0.05 and log2(fold change) ≥ 0.36 (Fig. 4A), and revealed a wide range of cellular structural alterations by GO pathway analysis (Fig. 4B).

Further analysis of the microglial population revealed eight subclusters (cluster 0–7, Fig. 4C). Subcluster 0 was the predominant microglial population in both CD47-overexpression and control mice, which has homeostatic microglial signatures with higher expression, including *P2ry12*, *Cst3*, *Hexb* and *Tmsb4x* [1, 20]. Compositional analysis showed that this subpopulation was significantly expanded, whereas subcluster 1, the second most common microglial population, was reduced in CD47-overexpression mice (Fig. 4D and Table S5). Furthermore, the enrichment analysis revealed that the downregulated genes in Cluster1 were enriched in the lytic vacuole and lysosome pathways (Fig S2E), indicating reduced phagocytic and lysosomal functions in these mice. Interestingly, subcluster 1 was enriched for DAM-like signatures, including *Apoe*, *Ctsb*, *Lyz2*, *Itgax*, and *Clec7a* [1, 20]. Neuronal CD47 overexpression significantly upregulated homeostatic microglial signatures (Fig. 4G and Table S6), while downregulating DAM-like signatures (Fig. 3H and Table S6). These findings reveal that CD47 neuronal overexpression shifts microglial signatures from DAM-like to homeostatic.

### Neuronal CD47 overexpression enhances behavioral disinhibition in 5xFAD mice

**Fig. 5:**
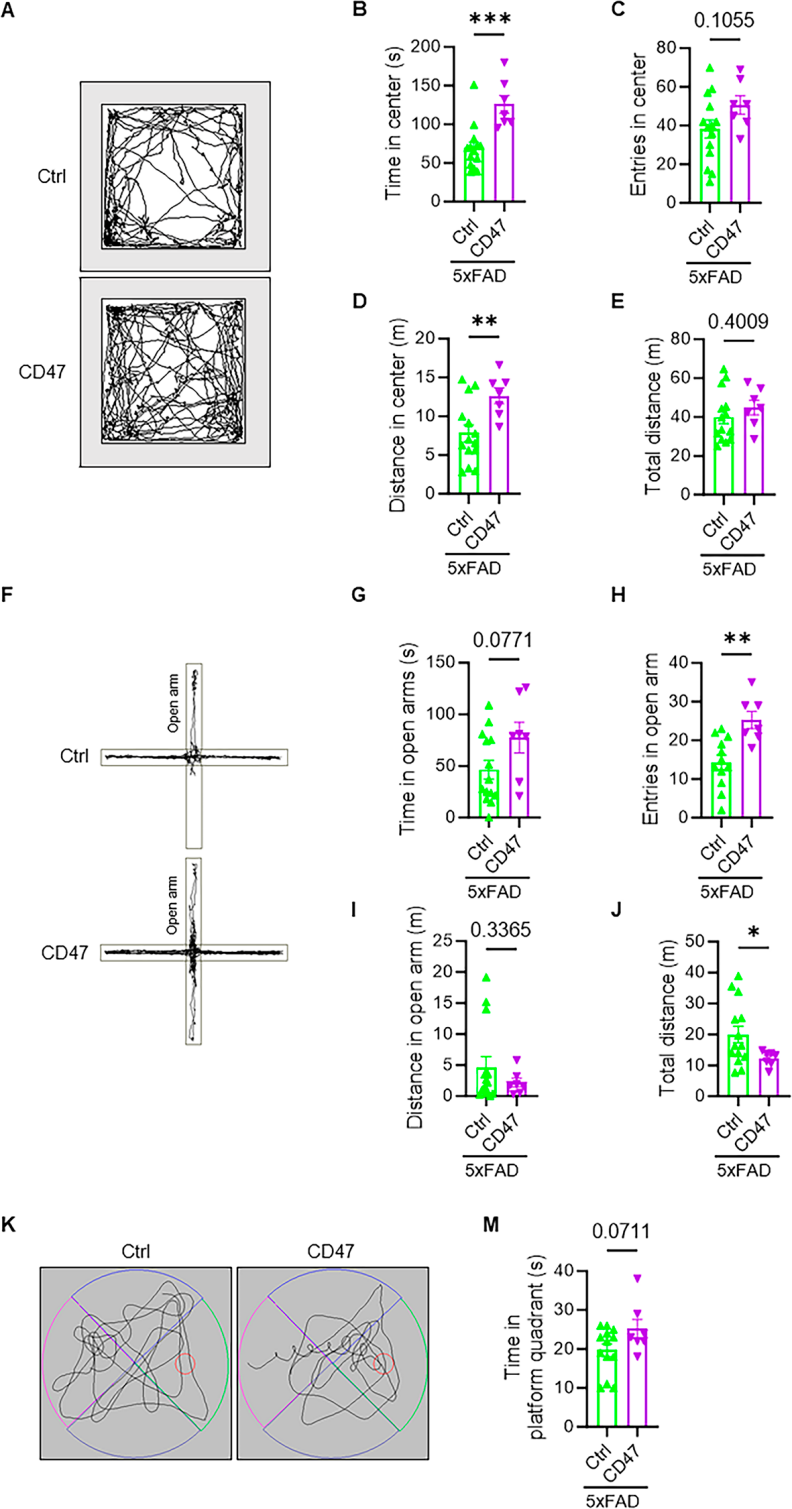
Behavior profiling in 5xFAD mice with neuronal CD47 overexpression. **(A)** A representative trajectory plot of control mice and mice with neuronal CD47 overexpression under 5xFAD background in an open field arena. **(B-E)** Time spent **(B)**, number of entries **(C)**, and distance traveled **(D)** in the inner zones of the open field arena (*n* = 14 for control, *n* = 7 for CD47 overexpression mice under 5xFAD background). **(E)** Total distance traveled in the inner and outer zones of the open field arena (*n* = 14 for control, *n* = 7 for CD47 overexpression mice under 5xFAD background). **(F)** A representative trajectory plot of control mice and 5xFAD mice with neuronal CD47 overexpression in the elevated plus maze. **(G-J)** Time spent **(G)**, number of entries **(H)**, and distance traveled **(I)** in the open arm of the elevated plus maze. **(J)** Total distance traveled in the open and closed arms of the elevated plus maze (*n* = 14 for control, *n* = 7 for CD47 overexpression mice under 5xFAD background). **(K)** A representative trajectory plot of control mice and 5xFAD mice with neuronal CD47 overexpression in the Morris water maze. **(M-N)** Time traveled and distance traveled in target quadrants of the Morris water maze (*n* = 14 for control, *n* = 7 for CD47 overexpression mice under 5xFAD background). Data are presented as the mean ± SEM. ^*^P ≤ 0.05, ^**^P ≤ 0.01, ^***^P ≤ 0.001

Since DAM is one of the most well-known disease-signature microglial populations in AD, we wondered whether CD47 overexpression in neurons could lead to behavioral alterations in an AD mouse model. Similar to wild-type mice, CD47-overexpression 5xFAD mice showed an increase in the time and distance in the center of the open field arena (Fig. 5A-B, D), and more entries in the open arms of the elevated plus maze (Fig. 5F, H). Although there was a trend of increased time in the platform quadrant in the Morris water maze test, the difference did not reach statistical significance (Fig. 5K, M). No significant differences were observed in escape latency or entries in the platform quadrant (Fig. S3A, B). These findings demonstrate that neuronal CD47 overexpression enhances behavioral disinhibition and memory performance in 5xFAD mice.

### Neuronal CD47 overexpression reduces synaptic loss in 5xFAD mice

**Fig. 6:**
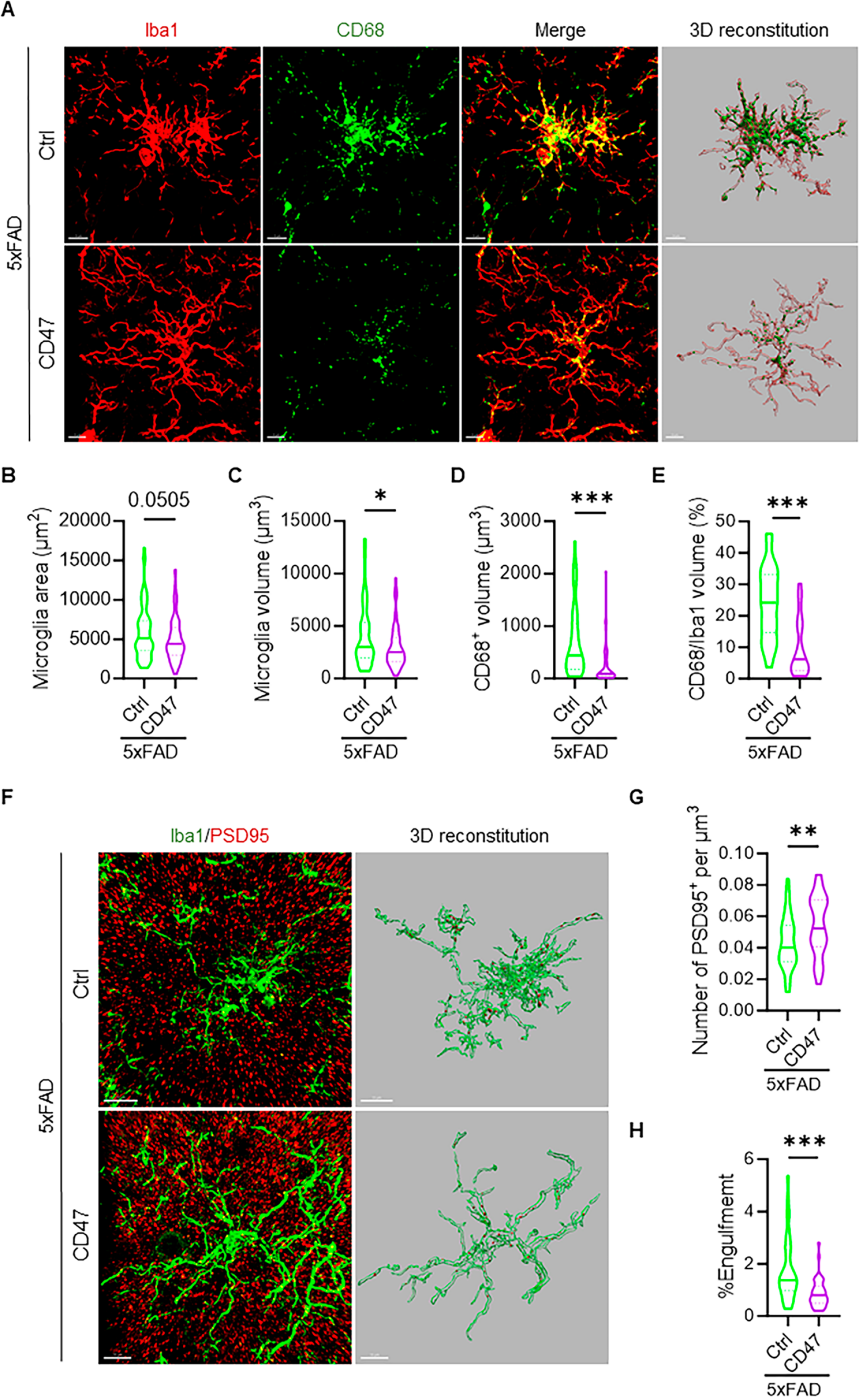
Neuronal CD47 overexpression reduces synaptic pruning in 5xFAD mice. **(A)** Representative images of Iba1 and CD68 co-immunostaining, along with 3D renderings, in the hippocampal CA3 region of control and neuronal CD47-overexpression mice under 5xFAD background at 4 months of age. Scale bar: 10µM. **(B-C)** Quantification of the surface area **(B)** and cellular volume **(C)** of microglia in the hippocampus of control and neuronal CD47 overexpression mice under 5xFAD background. **(D-E)** Quantification of CD68-positive volume **(D)** and the percentage of CD68-positive volume within microglia **(E)**. (*n* = 4 for control, *n* = 4 for CD47 overexpression mice under 5xFAD background). **(F)** Representative images of Iba1 and PSD95 co-immunostaining, along with 3D renderings, in the hippocampal CA3 region of control and neuronal CD47-overexpression mice under 5xFAD background at 4 months of age. Scale bar: 10µM. **(G-H)** Quantification of the number of PSD95 puncta **(G)** and the percentage of PSD95-positive volume within microglia **(H)**. (*n* = 4 for control, *n* = 4 for CD47 overexpression mice under 5xFAD background). Data are presented as the mean ± SEM. ^*^P ≤ 0.05, ^**^P ≤ 0.01, ^***^P ≤ 0.001

Microglia-mediated excessive synaptic pruning contributes to the pathogenesis of AD. We investigated whether neuronal CD47 overexpression could mitigate excessive synaptic phagocytosis by microglia in the CA3 region of the hippocampus in 4-month-old CD47-overexpressing and control mice. Immunofluorescent staining of Iba1 revealed a significant reduction in microglial surface area (Fig. 6A-B) and volume (Fig. 6A-C), indicating a less activated status due to CD47 neuronal overexpression. Additionally, CD47 neuronal overexpression also decreased phagocytosis, as indicated by a reduced microglial CD68 signal (Fig. 6A, D-E). Consistent with these findings, CD47 neuronal overexpression decreased the level of PSD95 puncta engulfed by microglia (Fig. 6, F, H) and increased total PSD95-positive signals (Fig. 6, F-G) in the hippocampus. These results demonstrate that neuronal CD47 expression mitigates microglial overactivation and excessive synaptic pruning in the 5xFAD mouse model.

Amyloid plaques and microglial overactivation are two major pathological hallmarks in the 5xFAD mouse model. We next sought to test whether they were altered by CD47 neuronal overexpression. Thioflavin S staining that recognizes amyloid plaques showed significantly lower fluorescence intensity in the hippocampus in CD47-overexpression mice than in control mice (Fig. S3 A, B). Consistent with this result, morphological analysis revealed a reduced volume and surface area of plaques in the hippocampus of CD47-overexpression mice (Fig. S3 C-E). These results demonstrate that neuronal CD47 expression reduces plaque pathology in the 5xFAD mouse model.

## Discussion

Beyond active immune defense, microglia play critical roles in neuronal function, normal brain development, and mechanisms of neuropsychiatric disorders. The DAM’s subpopulation, which have been identified through single-cell or single-nucleus transcriptome analysis, are known to expand under a variety of pathological conditions [1, 27–29]. However, the signals that trigger the expansion of DAM subpopulations remain unclear due to the complicated co-existing pathological hallmarks, which makes it difficult to dissect precise regulatory signals for DAM. Although termed disease-associated microglia, there are trivial subpopulations that express DAM signatures under physiological condition [1, 30]. Consistent with this, we identified a small portion of DAM-like microglia in wild-type mice by snRNA-seq analysis. The functions of DAM-like microglia under physiological conditions, as well as the associated regulatory signaling pathways, warrant further investigation. Here, our results provide mechanistic insights into this question. We utilized AAV-mediated neuronal gene overexpression of CD47 to upregulate “do not eat me” signals in neurons and observed that neuronal CD47 shifted microglial gene expression signatures from DAM-like to homeostatic states. This suggests that a low degree of synaptic pruning pathway activity acts as DAM triggering signal under physiological conditions, which is not surprising given DAM’s high level of phagocytic pathway [20, 31]. In accordance with these shifted gene expression signatures of microglia, neuronal CD47 reduces microglial phagocytosis and increases the levels of neuronal synaptic pathways and overall synapses. Furthermore, mice with neuronal CD47 overexpression exhibit ASD-like behaviors, indicating that DAM-like microglia play functional role in synaptic pruning under physiological conditions and are involved in the pathogenesis of ASD.

Consistent with decreased synaptic pruning, snRNA-seq analysis also shows that synaptic pathways are the most highly enriched pathways in different types of neurons, indicating enhanced synaptic connections. Additionally, CD47 was overexpressed mainly in the cortex and hippocampus, suggesting that refinement of neural circuits in these specific brain regions may also be involved in the profound behavioral alterations observed. Furthermore, it remains possible that CD47 functions in a cell-autonomous manner, as studies have shown that CD47 can promote neuronal development without the participation of microglia [32]. Moreover, SIRPα, a CD47 receptor mediating the “do not eat me” signal [33, 34], is expressed in cell types other than microglia such as neurons, astrocytes, and oligodendrocytes [35], which can lead to microglia-independent effects. Further investigations are required to decipher the underlying neural mechanisms and distinguish between the cell-autonomous and non-autonomous effects of neuronal CD47 overexpression.

We observed that neuronal CD47 overexpression led to ASD-like behaviors, which aligns with previous report that overexpression of CD47 is detrimental and associated with 16p11.2 deletion syndrome, one of the most severe forms of ASD [24]. Notably, we exclusively upregulated CD47 gene expression and examined its functions specifically in neurons, while 16p11.2 deletion probably leads to widespread CD47 overexpression across various cell types in the brain and peripheral systems. Furthermore, 16p11.2 deletion likely has broader direct functions beyond merely indirectly upregulating CD47 expression, as the deletion occurs on the short arm of chromosome 16 [36], while the CD47 gene is located on the third chromosome [37]. Nevertheless, our findings reveal a strong effect of neuronal CD47, which alone can trigger the ASD phenotype.

Interestingly, neuronal CD47 overexpression also promotes memory and cognitive performance, indicating beneficial effects that could offer a new perspective on the pathogenic mechanisms and therapeutic targets for AD. Microglial overactivation and synaptic pruning have been proven to correlate with cognitive impairments [16, 38]. However, the consequences of synaptic pruning are complex. Uncontrollable pruning is expected to contribute to synaptic loss and disease pathogenesis. In contrast, provocative data imply that pruning can be protective by reducing hyperexcitability at the early stage of the disease [39, 40]. In our study, neuronal CD47 overexpression reduces microglial engulfment of synapses and overall synaptic loss in 5xFAD mice, and shows beneficial trends in behavioral tests. These findings suggest that synaptic pruning-targeted therapies could be promising intervention strategies.

It is well-established that CD47, a key molecule acting as a “do not eat me” signal in synaptic pruning, protects synapses from microglial engulfment during development. Our work reveals that neuronal CD47 overexpression causes ASD-like behaviors and synaptic pruning deficits. Mechanistically, neuronal CD47 shifts microglial expression signatures from DAM-like to homeostatic states in wild-type mice, suggesting a functional role for DAM-like microglia under physiological conditions. Moreover, neuronal CD47 overexpression reduces microglial engulfment of synapses and overall synaptic loss caused by Aβ and plaque pathology, indicating that CD47 mediated pruning inhibition is a therapeutic target for AD.

### Limitations of the study

Our study has several limitations. First, we used an AAV-mediated gene expression system, thus the CD47 expression level varies between regions and individual cells. The creation of a genetic mouse model with uniform gene expression will be ideal in this regard. Second, whereas our study focused on neuron-microglia interactions, neuronal CD47 overexpression may also affect other cell types, such as astrocytes and oligodendrocytes, which may also contribute to the phenotypes observed in our study [41]. Third, microglia and synaptic pruning are highly dynamic processes and contribute to early synaptic loss in AD mouse model [16]. We analyzed 5xFAD mice at relatively early stage (4 months of age). Additional work at later time points will be helpful to better understand how microglial phagocytosis contributes to the pathogenesis of the disease. Last, the 5xFAD mouse model used in our study produces a much higher level of Aβ than that in the brains of human AD patients. Moreover, it is associated with only plaque pathology and lacks phosphorylated tau and neurofibrillary tangles pathology [42]. Therefore, the extent to which our findings can be generalized to human AD requires further investigation.

## Electronic supplementary material

Below is the link to the electronic supplementary material.

Supplementary Material 1: **Fig ****S1****. (A-B)** Representative immunofluorescence images of CD47 staining in control and neuronal CD47 overexpression mice at 2 months of age. Scale bar: 1000 μm. **(C)** Representative immunofluorescence images of CD47 and NeuN co-staining by confocal microscopy in control and neuronal CD47 overexpression at 4 months of age. Scale bar: 15 μm

Supplementary Material 2: **Fig ****S2****. (A)** UMAP plots of 21,721 cells from hippocampus of control and CD47 overexpression mice after batch effect corrections. **(B)** Violin plot showing the expression levels of CD47 gene in in various cell subpopulations. **(C)** Violin plot showing the expression levels of SIRPα gene in in various cell subpopulations. **(D)** Western blot image of CD47 protein expression in control and neuronal CD47-overexpression mice under wild-type and 5xFAD backgrounds at 4 months of age. **(E)** GO enrichment analysis for downregulated genes in Cluster 1 of CD47-overexpression versus control mice

Supplementary Material 3: **Fig ****S3****. (A-B)** The escape latency (A) and entries in platform quadrant (B) of control mice and 5xFAD mice with neuronal CD47 overexpression in the Morris water maze. **(C-D)** Representative images of Thioflavin S staining in the brains of control and CD47 overexpression 5xFAD mice by fluorescence microscopy (C) with quantification of the Thioflavin S positive area (D) (*n* = 15 brain slices from 5 mice for control, *n* = 6 brain slices from 3 mice for CD47 overexpression 5xFAD mice). Scale bar: 450 μm. **(E-G)** Representative 3D rendering of Thioflavin S and Iba1 immunostaining in the brains of control and CD47 overexpression 5xFAD mice by confocal microscopy (E) with quantification of Thioflavin S positive plaque volume (*n* = 46–66 plaques from 3–5 mice/group) (F), plaque surface area (G). Scale bar: 10 μm. Data are presented as the mean ± SEM. ^*^P ≤ 0.05, ^**^P ≤ 0.01

Supplementary Material 4

Supplementary Material 5

## Abbreviations

ASD: Autism spectrum disorder
AD: Alzheimer’s disease
DAM: Disease-associated microglia
MGnD: Microglial neurodegenerative phenotype
Aβ: Ayloid β
OFT: Open field test
EPM: Elevated plus maze
MWM: Morris water maze
snRNA-seq: Single-nucleus RNA sequencing
DEGs: Differentially expressed genes
GO: Gene Ontology
