# Supplementary figures and images for: Neuronal CD47 induces behavioral alterations and ameliorates microglial synaptic pruning in wild-type and Alzheimer’s mouse models
Source: Cell Biosci. 2025 Mar 26;15:38. doi: 10.1186/s13578-025-01378-x (PMC11948738; doi:10.1186/s13578-025-01378-x)

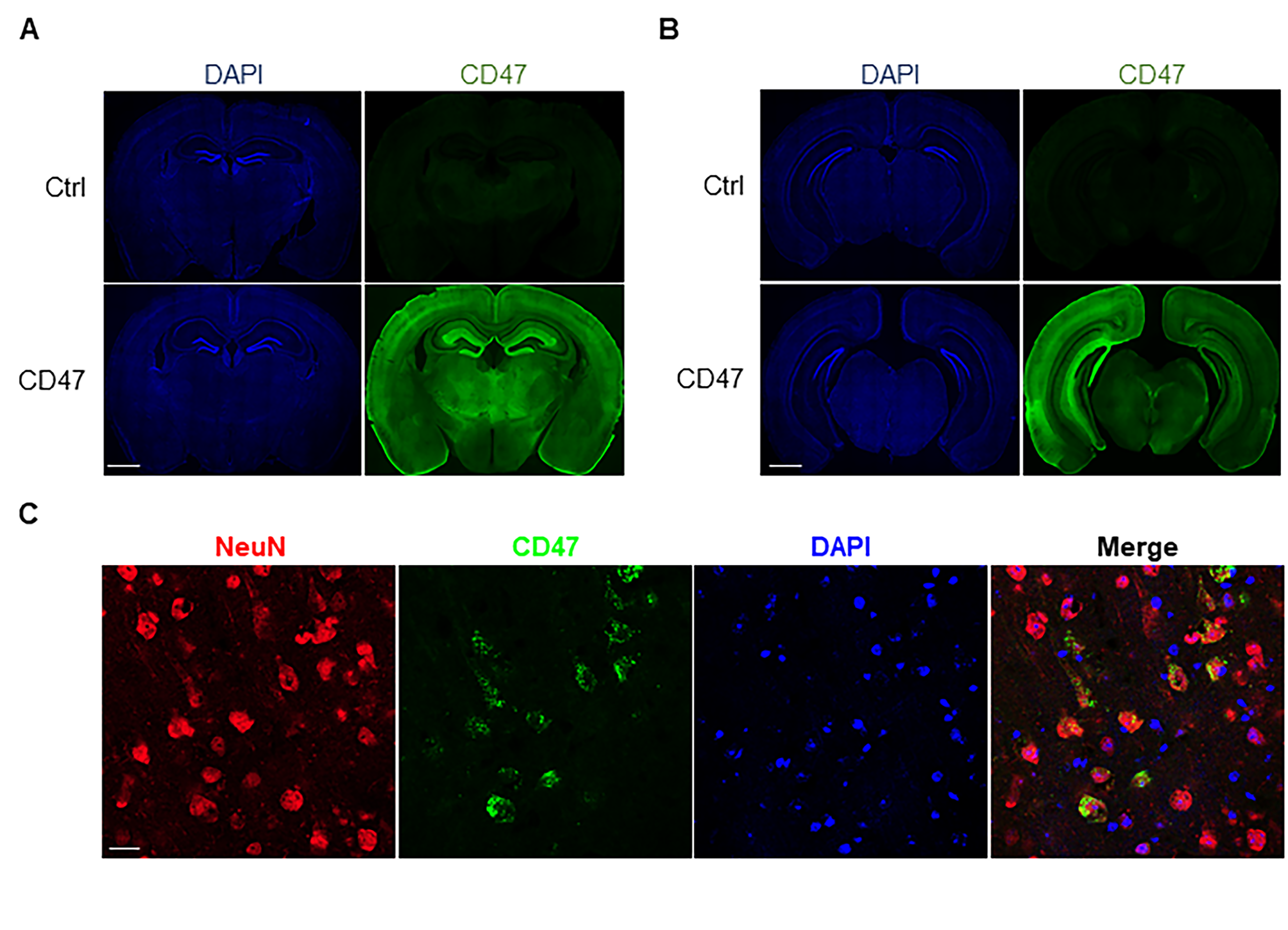

Supplement: Supplementary file 1 — Supplementary Material 1: Fig S1. (A-B) Representative immunofluorescence images of CD47 staining in control and neuronal CD47 overexpression mice at 2 months of age. Scale bar: 1000 μm. (C) Representative immunofluorescence images of CD47 and NeuN co-staining by confocal microscopy in control and neuronal CD47 overexpression at 4 months of age. Scale bar: 15 μm [file 13578_2025_1378_MOESM1_ESM.tif]

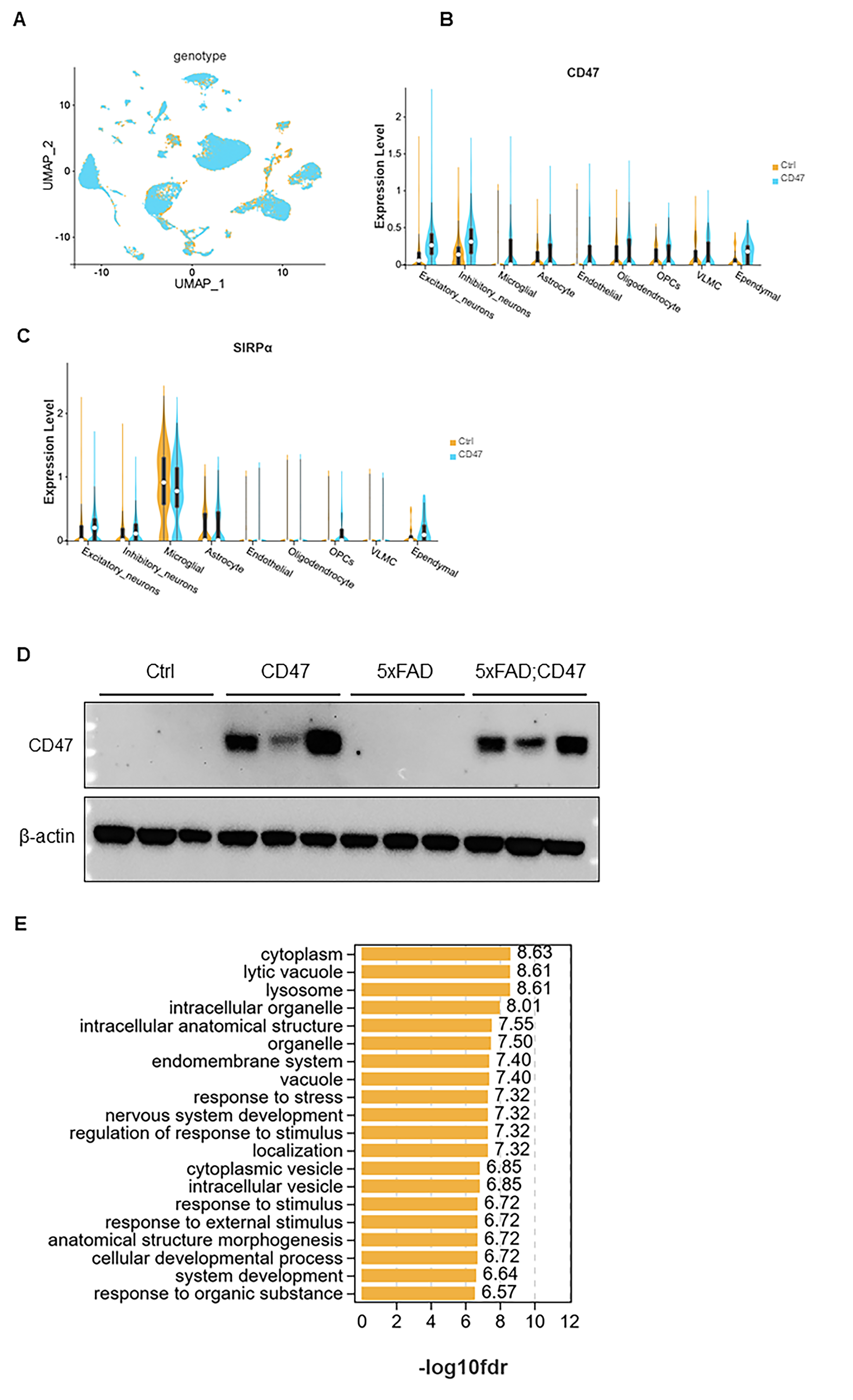

Supplement: Supplementary file 2 — Supplementary Material 2: Fig S2. (A) UMAP plots of 21,721 cells from hippocampus of control and CD47 overexpression mice after batch effect corrections. (B) Violin plot showing the expression levels of CD47 gene in in various cell subpopulations. (C) Violin plot showing the expression levels of SIRPα gene in in various cell subpopulations. (D) Western blot image of CD47 protein expression in control and neuronal CD47-overexpression mice under wild-type and 5xFAD backgrounds at 4 months of age. (E) GO enrichment analysis for downregulated genes in Cluster 1 of CD47-overexpression versus control mice [file 13578_2025_1378_MOESM2_ESM.tif]

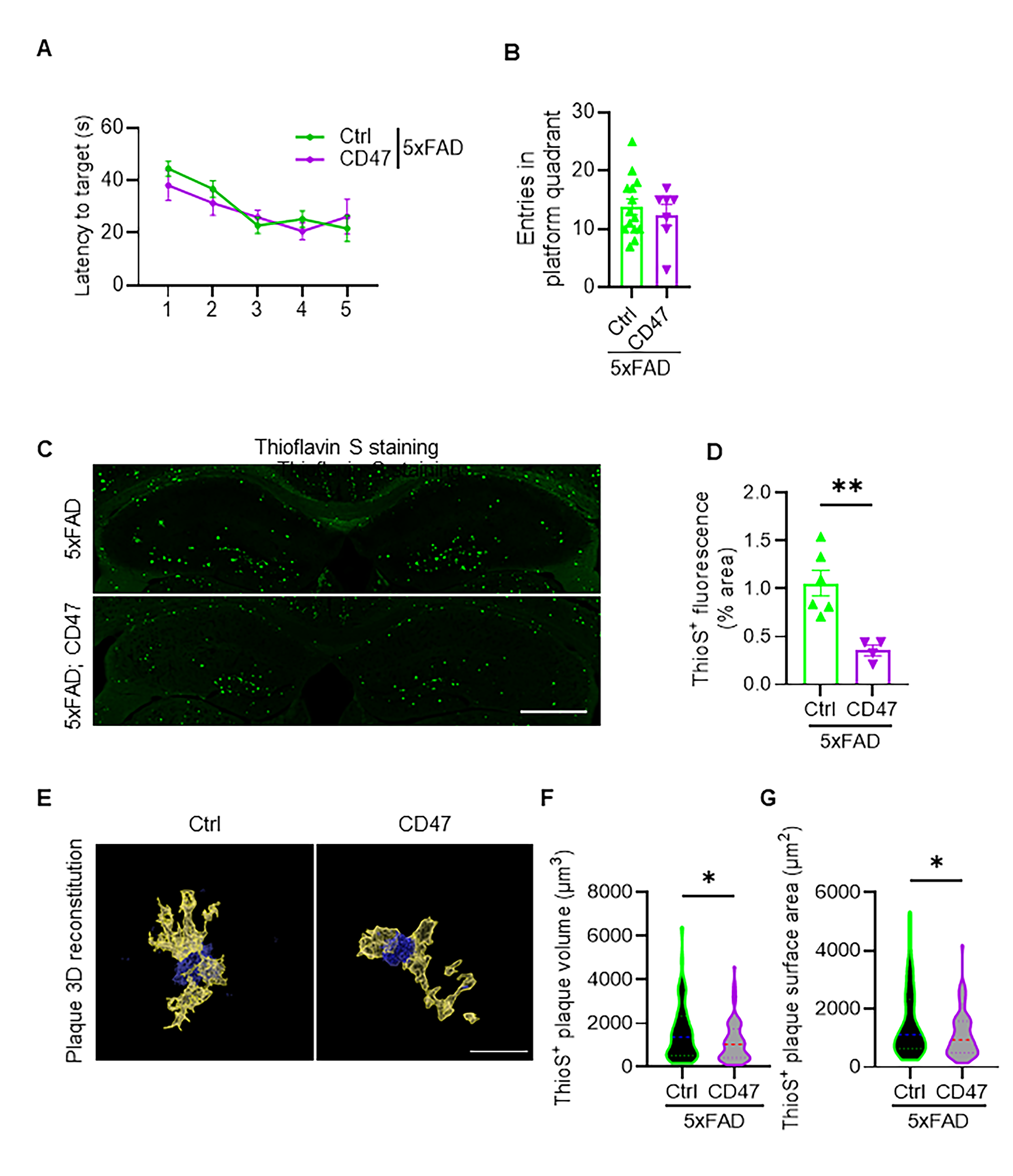

Supplement: Supplementary file 3 — Supplementary Material 3: Fig S3. (A-B) The escape latency (A) and entries in platform quadrant (B) of control mice and 5xFAD mice with neuronal CD47 overexpression in the Morris water maze. (C-D) Representative images of Thioflavin S staining in the brains of control and CD47 overexpression 5xFAD mice by fluorescence microscopy (C) with quantification of the Thioflavin S positive area (D) (n = 15 brain slices from 5 mice for control, n = 6 brain slices from 3 mice for CD47 overexpression 5xFAD mice). Scale bar: 450 μm. (E-G) Representative 3D rendering of Thioflavin S and Iba1 immunostaining in the brains of control and CD47 overexpression 5xFAD mice by confocal microscopy (E) with quantification of Thioflavin S positive plaque volume (n = 46–66 plaques from 3–5 mice/group) (F), plaque surface area (G). Scale bar: 10 μm. Data are presented as the mean ± SEM. *P ≤ 0.05, **P ≤ 0.01 [file 13578_2025_1378_MOESM3_ESM.tif]
