# Supplementary material for: Neuronal CD47 induces behavioral alterations and ameliorates microglial synaptic pruning in wild-type and Alzheimer’s mouse models
Source: Cell Biosci. 2025 Mar 26;15:38. doi: 10.1186/s13578-025-01378-x (PMC11948738; doi:10.1186/s13578-025-01378-x)
Supplement: Supplementary file 4 — Supplementary Material 4 [file 13578_2025_1378_MOESM4_ESM.docx]

**Supplementary Methods**

**1. Experiments**

**1.1 GEM generation & Barcoding**

Cellular suspensions were loaded on a 10X Genomics GemCode Single-cell instrument that generates single-cell Gel Bead-In-EMlusion (GEMs). Libraries were generated and sequenced from the cDNAs with Chromium Next GEM Single Cell 5’ Reagent Kits v3.1.

Upon dissolution of the Gel Bead in a GEM, primers containing (i) an Illumina® R1 sequence (read 1 sequencing primer), (ii) a 16 nt 10x Barcode, (iii) a 10 nt Unique Molecular Identifier (UMI), and (iv) a poly-dT primer sequence were released and mixed with cell lysate and Master Mix. Barcoded, full-length cDNAs were then reverse-transcribed from poly-adenylated mRNA.

**1.2 GEM-RT clean up & cDNA amplification**

Silane magnetic beads were used to remove leftover biochemical reagents and primers from the post GEM reaction mixture. Full-length, barcoded cDNAs were then amplified by PCR to generate sufficient mass for library construction.

**1.3 Library construction**

R1 (read 1 primer sequence) were added to the molecules during GEM incubation. P5, P7, a sample index, and R2 (read 2 primer sequence) were added during library construction via End Repair, A-tailing, Adaptor Ligation, and PCR. The final libraries contained the P5 and P7 primers used in Illumina bridge amplification.

**1.4 Sequencing**

The Single Cell 5’ Protocol produced Illumina-ready sequencing libraries. A Single Cell 5’ Library comprised standard Illumina paired-end constructs which begin and end with P5 and P7. The Single Cell 5’ 16 bp 10x Barcode and 10 bp UMI were encoded in Read 1, while Read 2 was used to sequence the cDNA fragment. Sample index sequences were incorporated as the i7 index read. Read 1 and Read 2 were standard Illumina® sequencing primer sites used in paired-end sequencing.

**2. Bioinformatic analysis**

**2.1 Data quality control and gene expression quantification**

10X Genomics Cell Ranger software (version 3.1.0) was used to convert raw BCL files to FASTQ files, alignment and counts quantification.

**2.1.1 Data quality control and genome alignment**

Briefly, reads with low-quality barcodes and UMIs were filtered out and then mapped to the reference genome. Reads uniquely mapped to the transcriptome and intersecting an exon at least 50% were considered for UMI counting.

**2.1.2 Gene expression quantification**

Before quantification, the UMI sequences would be corrected for sequencing errors, and valid barcodes were identified based on the EmptyDrops method.[1] The cell by gene matrices were produced via UMI counting and cell barcodes calling.

**2.2 Cell clustring**

The cell by gene matrices for each sample were individually imported to Seurat [2] version 3.1.1 for downstream analysis.

**2.2.1 Expression QC**

Cells with unusually high number of UMIs (≥8000) or mitochondrial gene percent (≥10%) were filtered out. We also excluded cells with less than 500 or more than 4000 genes detected. Additionally, doublet GEMs also should be filtered out. It was achieved by using the tool DoubletFinder (v2.0.3) by the generation of artificial doublets, using the PC distance to find each cell’s proportion of artificial k nearest neighbors (pANN) and ranking them according to the expected number of doublets[3].

**2.2.2 Normalizing the data**

After removing unwanted cells from the dataset, we employed a global-scaling normalization method “LogNormalize” that normalizes the gene expression measurements for each cell by the total expression, multiplies this by a scale factor (10,000 by default), and log-transforms the results. The formula is shown as follows:

$$A gene expression level=\log\left( 1+\frac{{UMI}_{A}}{{UMI}_{Total}}\times10000 \right)$$

**2.2.3 Batch effect correction**

To minimize the effects of batch effect and behavioral conditions on clustering, we used Harmony, an algorithm that projects cells into a shared embedding in which cells group by cell type rather than dataset-specific conditions, to aggregate all samples.[4] The Harmony algorithm inputs a PCA embedding of cells, along with their batch assignments, and returns a batch corrected embedding.

**2.2.4 PCA（Principal component analysis）**

Integrated expression matrix is then scaled and performed on principal component analysis for dimensional reduction. Then we implemented a resampling test inspired by the jackStraw procedure. We randomly permuted a subset of the data (1% by default) and rerun PCA, constructing a ‘null distribution’ of gene scores, and repeated this procedure. We identified ‘significant’ PCs as those who have a strong enrichment of low p-value genes for downstream clustering and dimensional reduction.[5]

**2.2.5 cells clustring**

Seurat implements a graph-based clustering approach. Distances between the cells were calculated based on previously identified PCs. Briefly, Seurat embed cells in a shared-nearest neighbor (SNN) graph, with edges drawn between cells via similar gene expression patterns. To partition this graph into highly interconnected quasi-cliques or communities, we first constructed the SNN graph based on the euclidean distance in PCA space and refined the edge weights between any two cells based on the shared overlap in their local neighborhoods (Jaccard distance). We then cluster cells using the Louvain[6] method to maximize modularity.

**2.2.6 clusters visualization**

For visualization of clusters, t-distributed Stochastic Neighbor Embedding (t-SNE) were generated using the same PCs.[7]

**2.2.7 Cell type annotation**

The log-normalized matrices were then loaded on SingleR R packages for cell type annotation, which based on correlating gene expression of reference cell types with single-cell expression. First, a Spearman coefficient is calculated for single-cell expression with each of the samples in the reference data set. Next, multiple correlation coefficients per cell type are aggregated to provide a single value per cell type per single cell. Finally, SinlgeR reruns the correlation analysis, but only for the top cell types from the previous step. The analysis was performed only on variable genes. The cell type corresponding to the top value after the last run is assigned to the single cell.[8]

**2.3 Differentially expressed genes (up-regulation) analysis**

**2.3.1 Differentially expressed genes analysis**

Expression value of each gene in given cluster were compared against the rest of cells using Wilcoxon rank sum test[9]. Significant upregulated genes were identified using a number of criteria. First, genes had to be at least 1.28-fold overexpressed in the target cluster. Second, genes had to be expressed in more than 25% of the cells belonging to the target cluster. Third, p value is less than 0.05.

**2.3.2 GO enrichment analysis**

Gene Ontology (GO) is an international standardized gene functional classification system which offers a dynamic-updated controlled vocabulary and a strictly defined concept to comprehensively describe properties of genes and their products in any organism. GO has three ontologies: molecular function, cellular component, and biological process. The basic unit of GO is GO-term. Each GO-term belongs to a type of ontology.[10]

GO enrichment analysis provides all GO terms that significantly enriched in differentially expressed genes comparing to the genome background and filter the differentially expressed genes that correspond to biological functions. Firstly all peak related genes were mapped to GO terms in the Gene Ontology database (http://www.geneontology.org/), gene numbers were calculated for every term, significantly enriched GO terms in differentially expressed genes comparing to the genome background were defined by hypergeometric test. The calculating formula of P-value is:

$$P=1-\sum_{i=0}^{m-1} \frac{\binom{M}{i}\binom{N-M}{n-i}}{\binom{N}{n}}$$

Here N is the number of all genes with GO annotation; n is the number of differentially expressed genes in N; M is the number of all genes that are annotated to the certain GO terms; m is the number of differentially expressed genes in M. The calculated p-value were gone through FDR Correction, taking FDR ≤ 0.05 as a threshold. GO terms meeting this condition were defined as significantly enriched GO terms in differentially expressed genes. This analysis was able to recognize the main biological functions that differentially expressed genes exercise.

**2.3.3 Pathway enrichment analysis**

Genes usually interact with each other to play roles in certain biological functions. Pathway- based analysis helps to further understand genes biological functions. KEGG is the major public pathway-related database.[11] Pathway enrichment analysis identified significantly enriched metabolic pathways or signal transduction pathways in differentially expressed genes comparing with the whole genome background.[10] The calculating formula is the same as that in GO analysis:

$$P=1-\sum_{i=0}^{m-1} \frac{\binom{M}{i}\binom{N-M}{n-i}}{\binom{N}{n}}$$

Here N is the number of all transcripts that with KEGG annotation, n is the number of

differentially expressed genes in N, M is the number of all transcripts annotated to specific pathways, and m is number of differentially expressed genes in M. The calculated p-value was gone through FDR Correction, taking FDR ≤ 0.05 as a threshold. Pathways meeting this condition were defined as significantly enriched pathways in differentially expressed genes.

**3. Reference**

[1] Lun ATL, Riesenfeld S, Andrews T, et al. EmptyDrops: distinguishing cells from empty droplets in droplet-based single-cell RNA sequencing data. Genome Biol. 2019;20(1):63.

[2] Butler A, Hoffman P, Smibert P, et al. Integrating single-cell transcriptomic data across different conditions, technologies, and species. Nat Biotechnol.2018;36(5):411-420.

[3] McGinnis CS, Murrow LM, Gartner ZJ. DoubletFinder: Doublet Detection in Single-Cell RNA Sequencing Data Using Artificial Nearest Neighbors. Cell Syst. 2019 Apr 24;8(4):329-337.e4. doi: 10.1016/j.cels.2019.03.003.

[4] Korsunsky I, Millard N, Fan J, Slowikowski K, Zhang F, Wei K, Baglaenko Y, Brenner M, Loh PR, Raychaudhuri S. Fast, sensitive and accurate integration of single-cell data with Harmony. Nat Methods. 2019 Dec;16(12):1289-1296. doi: 10.1038/s41592-019-0619-0.

[5] Chung NC, Storey JD. Statistical significance of variables driving systematic variation in high-dimensional data [J]. Bioinformatics. 2015;31(4):545-554.

[6] R. Rotta, A. Noack. Multilevel Local Search Algorithms for Modularity Clustering. J. Exp. Alg orithmics. 2011;16, 2.3.

[7] van der Maaten Laurens & Hinton Geoffrey. Visualizing data using t-SNE. Journal of Machine Learning Research. 2008;9(November):2579–2605.

[8] Aran D, Looney AP, Liu L, et al. Reference-based analysis of lung single-cell sequencing reveals a transitional profibrotic macrophage. Nat Immunol. 2019;20(2):163-172.

[9] Camp JG, Sekine K, Gerber T, et al. Multilineage communication regulates human liver bud development from pluripotency. Nature. 2017;546(7659):533-538.

[10] Ashburner M, Ball CA, Blake JA, et al. Gene ontology: tool for the unification of

biology. The Gene Ontology Consortium [J]. Nature Genetics, 2000;25(1):25-29.

[11] Kanehisa M, Goto S. KEGG: kyoto encyclopedia of genes and genomes [J]. Nucleic Acids Research, 2000;28(1):27-30.
