# Supplementary material for: Neuronal CD47 induces behavioral alterations and ameliorates microglial synaptic pruning in wild-type and Alzheimer’s mouse models
Source: Cell Biosci. 2025 Mar 26;15:38. doi: 10.1186/s13578-025-01378-x (PMC11948738; doi:10.1186/s13578-025-01378-x)
Supplement: Supplementary file 5 — Supplementary Material 5 [file 13578_2025_1378_MOESM5_ESM.docx]

**Table S1: Antibody list.**

| **Antibody** | **Source** | **Company** | **Catalog Number** |
| --- | --- | --- | --- |
| FLAG | Mouse | Sigma | F1804 |
| β-Actin | Mouse | Proteintech | 66009 |
| CD47 | Rat | BD PharMingen | 555297 |
| Iba1 | Goat | Novus Biologicals | NB100-1028 |
| PSD95 | Rabbit | CST | #3405 |
| CD68 | Rat | Bio-Rad | MCA1957GA |
| HRP-Donkey Anti-Rat | Donkey | Proteintech | SA00001-15 |
| HRP-Goat Anti-Mouse | Goat | Proteintech | SA00001-1 |
| Alexa Fluor® 488 | Donkey | Abcam | ab150165 |
| Alexa Fluor® 594 | Donkey | Abcam | ab150080 |

**Table S2: A summary of the single nucleus RNA-seq data generated in this study (related to Figure 3).**

| **Sample** | **Genotype** | **Age** | **Cell numbers (before filtering)** | **Cell numbers (after filtering)** | **Mean UMIs** | **Mean gene numbers** |
| --- | --- | --- | --- | --- | --- | --- |
| Ctrl_1 | C57/BL6 | 4M | 12,777 | 11,468 | 4,648.5 | 2,338 |
| Ctrl_2 | C57/BL6 | 4M |  |  |  |  |
| CD47_1 | C57/BL6 | 4M | 11,351 | 10,263 | 6,712 | 3,135 |
| CD47_2 | C57/BL6 | 4M |  |  |  |  |

**Table S3: The expression levels of CD47 and SIRPα genes in Ctrl and CD47 respectively. (related to Figure S2, C and D).**

| **Cluster** | **Gene ID** | **Gene Name** | **Ctrl** | **CD47** | **cells1** | **cells2** | **pct1** | **pct2** | **log2FC** | **p_value** | **FDR** | **significance** |
| --- | --- | --- | --- | --- | --- | --- | --- | --- | --- | --- | --- | --- |
| Excitatory_neurons | ENSMUSG00000055447 | Cd47 | 1.443654173 | 4.519431179 | 7526 | 6169 | 0.509 | 0.87 | 1.64634801 | 0 | 0 | up |
| Inhibitory_neurons | ENSMUSG00000055447 | Cd47 | 2.286157728 | 4.598470932 | 542 | 871 | 0.67 | 0.839 | 1.008197547 | 5.89E-36 | 3.35E-31 | up |
| Microglial | ENSMUSG00000055447 | Cd47 | 1.221317663 | 2.073579607 | 560 | 536 | 0.227 | 0.31 | 0.763636395 | 0.000175789 | 1 | up |
| Astrocyte | ENSMUSG00000055447 | Cd47 | 1.107938084 | 1.881351715 | 417 | 573 | 0.317 | 0.428 | 0.763838796 | 1.00E-06 | 0.057071418 | up |
| Endothelial | ENSMUSG00000055447 | Cd47 | 0.950885856 | 2.072791098 | 82 | 144 | 0.22 | 0.347 | 1.124148535 | 0.078223398 | 1 | nosig |
| Oligodendrocyte | ENSMUSG00000055447 | Cd47 | 1.513639173 | 2.548077417 | 1785 | 1334 | 0.357 | 0.489 | 0.751349087 | 3.33E-20 | 1.89E-15 | up |
| OPCs | ENSMUSG00000055447 | Cd47 | 0.946222145 | 1.621814042 | 184 | 239 | 0.326 | 0.444 | 0.77729407 | 0.001444772 | 1 | up |
| VLMC | ENSMUSG00000055447 | Cd47 | 1.289007116 | 1.756685797 | 89 | 142 | 0.281 | 0.366 | 0.446566149 | 0.375999106 | 1 | nosig |
| Ependymal | ENSMUSG00000055447 | Cd47 | 0.810812361 | 2.164242972 | 38 | 29 | 0.395 | 0.724 | 1.416311225 | 0.00919673 | 1 | up |
| Excitatory_neurons | ENSMUSG00000037902 | Sirpa | 1.255085976 | 1.68564512 | 7526 | 6169 | 0.453 | 0.681 | 0.425485283 | 3.39E-159 | 1.93E-154 | up |
| Inhibitory_neurons | ENSMUSG00000037902 | Sirpa | 1.277017698 | 1.224213508 | 542 | 871 | 0.443 | 0.521 | -0.060918455 | 1 | 1 | nosig |
| Microglial | ENSMUSG00000037902 | Sirpa | 10.61145807 | 8.753618714 | 560 | 536 | 0.827 | 0.8 | -0.277668568 | 1 | 1 | nosig |
| Astrocyte | ENSMUSG00000037902 | Sirpa | 1.749990743 | 2.09369632 | 417 | 573 | 0.432 | 0.469 | 0.258691379 | 1 | 1 | nosig |
| Endothelial | ENSMUSG00000037902 | Sirpa | 0.454335381 | 0.76106306 | 82 | 144 | 0.122 | 0.153 | 0.744130386 | 0.671527263 | 1 | nosig |
| Oligodendrocyte | ENSMUSG00000037902 | Sirpa | 0.380483779 | 0.474094591 | 1785 | 1334 | 0.111 | 0.135 | 0.317265133 | 1 | 1 | nosig |
| OPCs | ENSMUSG00000037902 | Sirpa | 0.724945491 | 0.841474027 | 184 | 239 | 0.239 | 0.259 | 0.215018664 | 1 | 1 | nosig |
| VLMC | ENSMUSG00000037902 | Sirpa | 0.613631158 | 0.867770789 | 89 | 142 | 0.135 | 0.183 | 0.499873437 | 0.522436112 | 1 | nosig |
| Ependymal | ENSMUSG00000037902 | Sirpa | 0.504944141 | 1.288153476 | 38 | 29 | 0.263 | 0.517 | 1.350935096 | 0.09038086 | 1 | nosig |

**Table S4: A summary of cell proportion of the single-cell cluster (related to Figure 3).**

| **Cluster** | **Ctrl** | **CD47** |
| --- | --- | --- |
| Excitatory_neurons | 7,526(65.63%) | 6,169(60.11%) |
| Oligodendrocyte | 1,785(15.57%) | 1,334(13.00%) |
| Microglial | 5,60(4.88%) | 536(5.22%) |
| Inhibitory_neurons | 542(4.73%) | 871(8.49%) |
| Astrocyte | 417(3.64%) | 573(5.58%) |
| OPCs | 184(1.6%) | 239(2.33%) |
| Unknown | 245(2.14%) | 226(2.20%) |
| Endothelial | 82(0.72%) | 144(1.40%) |
| VLMC | 89(0.78%) | 142(1.38%) |
| Ependyma | 38(0.33%) | 29(0.28%) |

**Table S5: A summary of cell proportion of the single-cell cluster (related to Figure 4).**

| **Cluster** | **Ctrl** | **CD47** |
| --- | --- | --- |
| 0 | 56.07% | 59.33% |
| 1 | 28.93% | 25.37% |
| 2 | 4.47% | 5.42% |
| 3 | 3.04% | 3.54% |
| 4 | 4.82% | 3.36% |
| 5 | 0.36% | 0.93% |
| 6 | 0% | 0.19% |
| 7 | 1.43% | 0.93% |

**Table S6: The expression levels of homeostatic and DAM genes in subcluster 0 and 1 microglia respectively. (related to Figure 4, G and H).**

| **Cluster** | **Gene ID** | **Gene Name** | **Ctrl** | **CD47** | **cells_1** | **cells_2** | **pct_1** | **pct_2** | **log2FC** | **p_value** | **FDR** | **significance** |
| --- | --- | --- | --- | --- | --- | --- | --- | --- | --- | --- | --- | --- |
| Cluster0 | ENSMUSG00000049775 | Tmsb4x | 24.98115017 | 49.54082343 | 167 | 146 | 0.976 | 1 | 0.987775083 | 9.61E-20 | 5.46E-15 | up |
| Cluster1 | ENSMUSG00000049775 | Tmsb4x | 21.16657766 | 42.84817697 | 309 | 308 | 0.984 | 1 | 1.017442351 | 3.70E-52 | 2.11E-47 | up |
| Cluster0 | ENSMUSG00000054675 | Tmem119 | 2.433709089 | 3.412491757 | 167 | 146 | 0.305 | 0.384 | 0.487651832 | 0.232438576 | 1 | nosig |
| Cluster1 | ENSMUSG00000054675 | Tmem119 | 2.366069303 | 3.827306593 | 309 | 308 | 0.362 | 0.519 | 0.693813865 | 0.000172631 | 1 | up |
| Cluster0 | ENSMUSG00000036353 | P2ry12 | 12.2176539 | 13.14405938 | 167 | 146 | 0.796 | 0.842 | 0.105442793 | 1 | 1 | nosig |
| Cluster1 | ENSMUSG00000036353 | P2ry12 | 10.39636392 | 15.2781604 | 309 | 308 | 0.777 | 0.916 | 0.555387368 | 2.63E-09 | 0.000149452 | up |
| Cluster0 | ENSMUSG00000021665 | Hexb | 15.73417717 | 20.83548973 | 167 | 146 | 0.886 | 0.904 | 0.405139032 | 0.011030242 | 1 | up |
| Cluster1 | ENSMUSG00000021665 | Hexb | 18.37712206 | 22.96666795 | 309 | 308 | 0.932 | 0.974 | 0.321629142 | 1 | 1 | nosig |
| Cluster0 | ENSMUSG00000027447 | Cst3 | 27.74625728 | 45.68189169 | 167 | 146 | 0.928 | 0.945 | 0.719327175 | 3.58E-06 | 0.203847463 | up |
| Cluster1 | ENSMUSG00000027447 | Cst3 | 24.35808262 | 54.54325355 | 309 | 308 | 0.903 | 0.994 | 1.162996909 | 1.41E-30 | 8.00E-26 | up |
| Cluster0 | ENSMUSG00000002985 | Apoe | 6.077809637 | 1.765534792 | 167 | 146 | 0.449 | 0.192 | -1.783388263 | 1.59E-06 | 0.090491635 | down |
| Cluster1 | ENSMUSG00000002985 | Apoe | 11.88371234 | 2.730288207 | 309 | 308 | 0.54 | 0.263 | -2.121819735 | 1.35E-17 | 7.68E-13 | down |
| Cluster0 | ENSMUSG00000021939 | Ctsb | 8.144365909 | 4.573298959 | 167 | 146 | 0.647 | 0.479 | -0.832553316 | 0.002435753 | 1 | down |
